# Role of mitochondria in neuronal function and survival in the enteric and central nervous systems

**DOI:** 10.1007/s00018-025-06053-5

**Published:** 2026-02-26

**Authors:** Irem Kural, Lobke Marie M Mombeek, David M. Wilson

**Affiliations:** Faculty of Medicine and Life Sciences, Biomedical Research Institute, Hasselt University, Diepenbeek, Belgium

**Keywords:** Mitochondrial dysfunction, Resilience mechanisms, Neuronal function, Central nervous system, Enteric nervous system

## Abstract

Mitochondria are indispensable organelles that not only generate cellular energy through oxidative phosphorylation but also regulate calcium homeostasis, redox balance, and apoptotic signaling. Given the high metabolic demands of neurons, mitochondrial function and resilience mechanisms are essential for neuronal development, maturation, and survival; when these systems fail, pathological outcomes can arise. This review highlights the critical role of mitochondria in maintaining neuronal function, with discussion related to both the central (CNS) and enteric (ENS) nervous systems. We present how mitochondrial dysfunction, through impaired bioenergetics, oxidative stress, defective quality control, and altered dynamics, can drive neuronal cell loss. Furthermore, we highlight the link between mitochondrial defects and nervous system pathological outcomes in both primary mitochondrial disorders, such as mitochondrial neurogastrointestinal encephalomyopathy, and secondary mitochondrial disorders, such as Alzheimer, Parkinson, and Huntington disease, as well as amyotrophic lateral sclerosis. By integrating evidence from the CNS and ENS, this review highlights the central role of mitochondria in supporting and preserving neuronal health, as well as the potential of mitochondria as therapeutic targets in neurodegenerative disease.

**Graphical abstract:**

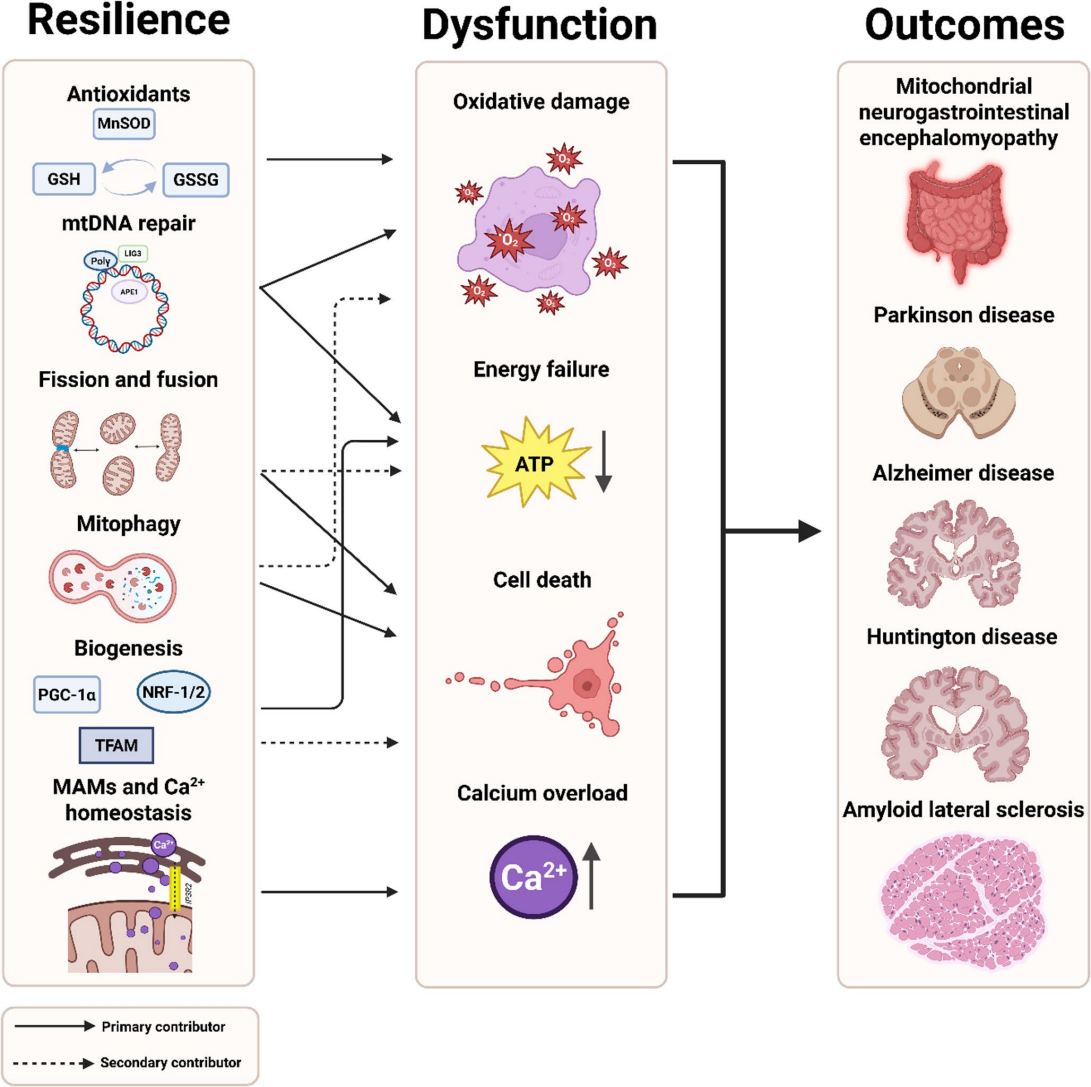

## Introduction

The regulation and maintenance of neuronal function constitute a major challenge for both the central (CNS) and enteric nervous systems (ENS) within the human body. The CNS, consisting of the brain and spinal cord, acts as the main processing center, receiving, interpreting, and responding to information from both inside and outside the body [1]. Despite accounting for only ~ 2% of total body weight, the CNS consumes about 20% of the body’s energy, indicating a reliance on efficient energy metabolism [2]. With an estimated 86 billion neurons and a comparable number of glia, which are collectively organized into specialized circuits and regions, the CNS regulates both behavior and physiology. This complex organization not only underpins higher cognitive functions such as memory, learning, and decision-making but also orchestrates essential autonomic processes, including respiration, cardiovascular regulation, and thermoregulation [3, 4].

The ENS, a complex network of ganglionated enteric neurons and glial cells embedded within the intestinal wall, functions semi-autonomously from the brain to orchestrate gastrointestinal (GI) activity. It is organized into two major plexus layers. The submucosal plexus, situated between the submucosa and the circular muscle layer, primarily regulates secretion of electrolytes, water, and mucus, as well as blood flow, while the myenteric plexus, located between the circular and longitudinal muscle layers, is mainly responsible for coordinating gut motility. Within these layers, distinct ganglia house diverse neuronal subtypes that assemble into neural circuits, enabling highly precise control of GI function [5]. Equally important, enteric glia, located within and around ENS ganglia, actively support neuronal health by maintaining homeostasis, regulating synaptic activity, modulating immune responses, and facilitating repair after injury [6].

It is generally known that neurons are highly specialized cells with complex morphologies and demanding functional requirements. They serve essential roles in receiving sensory input, sending motor commands, and relaying signals throughout the nervous system. Their exceptionally high metabolic cost arises from the maintenance of excitable membranes with voltage-gated ion channels, which support rapid, propagating shifts in membrane potential, which is the defining feature of action potentials [7]. Unlike many other cell types, neurons have a limited glycolytic capacity and rely predominantly on mitochondria for oxidative phosphorylation to meet their substantial energy demands [8]. Moreover, mitochondria are strategically distributed throughout neuron compartments, including the soma, dendrites, and axons, to support its diverse cellular energy needs [9, 10].

It is important to mention that recent work highlights that neuronal metabolism is more dynamic than initially believed. In particular, studies of triglyceride metabolism have shown that acute inhibition of the neuron-specific triglyceride lipase DDHD domain containing 2 (DDHD2) or the mitochondrial lipid transporter carnitine palmitoyltransferase 1 (CPT1) induces rapid torpor (i.e., hypometabolic adaptation) in mice, indicating that neurons continuously flux fatty acids from lipid droplets through β-oxidation to sustain mitochondrial ATP production [11]. Additionally, another study showed that neurons can indeed metabolize glucose through glycolysis in vivo and require this activity for optimal function [12]. These findings emphasize that while mitochondria remain central in neuronal energy production, neurons can flexibly utilize lipid and glucose pathways to maintain bioenergetic homeostasis.

The appearance of neurodegenerative hallmarks (i.e., pathological molecular processes) can drive neuronal cell loss, the main feature of neurodegenerative disease [13]. Most relevant to the discussion herein, “altered energy homeostasis”, commonly arising from mitochondrial dysfunction, can result in severely reduced ATP availability. In this review, we exhibit evidence for the indispensable role of mitochondria in fulfilling energy requirements within neuronal cells, concentrating mostly on studies related to the CNS, while highlighting early investigations relevant to the ENS. In addition, we present classic mitochondrial disorders to underscore how mitochondrial dysfunction critically impairs CNS neurons, and, based on emerging evidence, argue that similar vulnerabilities may extend to the ENS.

### The function of mitochondria in the formation of mature neurons

Mature neurons arise from neural progenitor cells (NPCs) in the developing CNS. During this process, mitochondria play key roles in neuronal differentiation by supporting both the bioenergetic shift and structural maturation of neurons. The bioenergetic shift entails the transition from glycolysis, which primarily fuels NPCs in the early stages, toward mitochondrial oxidative phosphorylation as neurons mature [14–16]. This metabolic remodeling is evidenced by (i) transcriptomic analyses during neurogenesis that reveal decreased expression of glycolytic genes, such as hexokinase 2 (*HK2)* and L-Lactate Dehydrogenase A *(LDHA)*, and (ii) mitochondrial DNA (mtDNA) copy number measurements, which indicate an increase in mitochondrial mass [17]. In addition, mitochondria are highly dynamic organelles that travel along microtubules to meet local energy demands. Their positioning in developing axons is, therefore, crucial for shaping neuronal architecture, including axonal and dendritic growth, as well as synapse formation [18, 19]. Thus, mitochondria are not only indispensable for meeting neuronal energy demands but also play a fundamental role in orchestrating the formation and maturation of neurons.

Within the forming ENS, mature neurons and glia arise from vagal and sacral neural crest–derived progenitor cells that migrate, proliferate, and differentiate along the developing gut. Although mitochondrial involvement during enteric neuronal development is understudied, Viader et al. reported that targeted deletion of the mitochondrial transcription factor A gene (*Tfam*) in enteric neurons led to profound GI defects [20]. This was done using a constitutive Cre-loxP approach with Cre expression under the control of the *Cnp* promoter to achieve recombination in gut neural crest stem cells and subsequently in the majority of enteric neurons and glia. Specifically, these Tfam-ENSKO (ENS knockout) mice exhibited premature death beginning at 60 days old due to chronic intestinal pseudo-obstruction (CIPO). CIPO is a severe clinical condition that mimics a mechanical bowel obstruction without a physical blockage and stems from defects in enteric neurons, smooth muscle, and/or autonomic innervation of the bowel [20]. This finding underscores that mitochondrial homeostasis is not only critical for neuronal development in the CNS but also indispensable for maintaining ENS integrity and function.

### Role of mitochondria in preserving mature neuronal cell viability

**Fig. 1:**
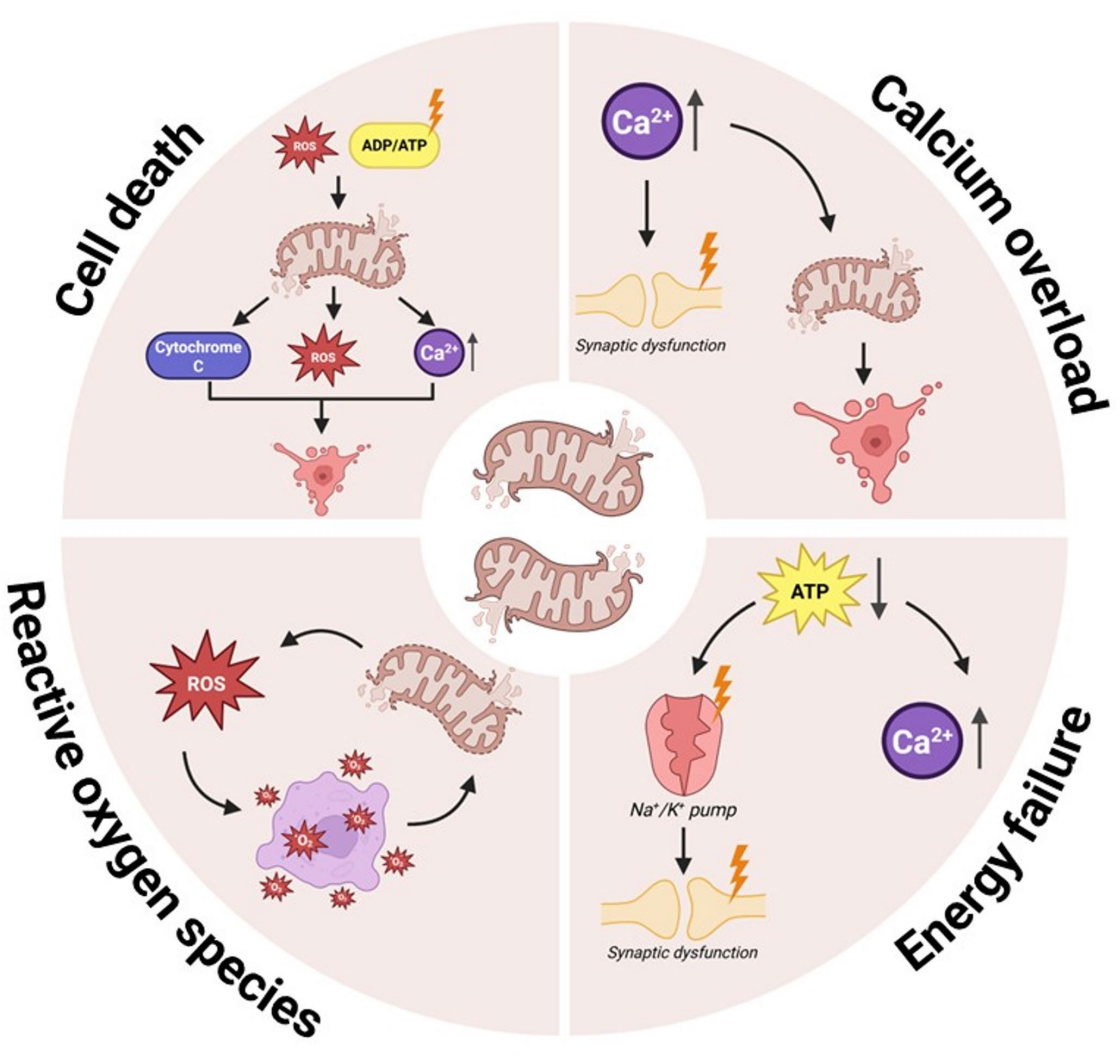
***Overview of mitochondrial dysfunction pathways.*** ADP, adenosine diphosphate; ATP, adenosine triphosphate; ROS, reactive oxygen species

As mentioned earlier, mature differentiated neurons are highly dependent on mitochondrial function due to their exceptional metabolic demands. A large fraction of the produced ATP by mitochondria is specifically required for the generation of action potentials along the axons [21]. In addition to their prominent role in generating ATP, mitochondria contribute in key ways to calcium homeostasis, redox balance, and apoptotic signaling. Together, these functions highlight mitochondria as critical organelles in mature neurons. In the following sub-sections, we review how specific molecular consequences (designated below) contribute to neuronal vulnerability and consequent cell loss. Figure 1 summarizes the pathways discussed.

#### Energy failure

Neurons are highly dependent on energy for their function, making them exceptionally vulnerable to disruptions in mitochondria and energy failure [7]. Energy failure is described as insufficient ATP production to sustain cellular functions and/or counteract stress and can arise from either impaired energy production, increased energy consumption, or both. Even relatively mild ATP deficits can impair neuronal function by disrupting ion gradients and synaptic transmission, yet may still be compatible with neuronal survival. More severe or prolonged deficits, however, can push neurons towards active forms of cell death, such as apoptosis [22]. In cases of extreme bioenergetic failure, there may be insufficient energy for classic apoptotic pathways to proceed, and energy failure may trigger other cell death pathways, such as necrosis [23].

Although it remains challenging to obtain direct evidence, since most available tools, techniques, and model systems lack the resolution to clearly establish causality, many neurodegenerative diseases are strongly associated with energy failure, which ultimately contributes to neuronal death (discussed in more detail later). Mitochondrial dysfunction is consistently observed in various neurodegenerative diseases, a phenotype that is likely responsible for the observed neuronal energetic failure [24–31]. A study by Le Masson et al. [32] explicitly explored the link between bioenergetics and motor neuron degeneration using a computational model in which detailed morphology and ion conductance are paired with intracellular ATP production and consumption. The investigators found that when ATP availability is reduced, each action potential becomes metabolically more demanding because the ion gradients become more difficult to restore. Due to this, the Na^+^/K^+^ homeostasis breaks down, resulting in chronic depolarization. Moreover, when ATP depletion is localized to distal axonal regions, local ionic imbalances can propagate along the axon, inducing twitching-like spiking activity and, eventually, whole-cell dysfunction. Additionally, ATP shortage was shown to increase intracellular calcium levels, compromising mitochondrial function and potentially triggering cell death [32].

While energy failure in the CNS has been more or less established, far less is known about its role in the ENS. Given the structural and functional similarities between CNS and ENS neurons, it is likely that energy failure similarly contributes to enteric neurodegeneration. Most notably, mitochondrial disorders with GI involvement, such as CIPO (see more later), may involve energy deficits in enteric neurons [33]. Although direct measurements of ATP levels or bioenergetic stress in the intact ENS are currently limited, Desmet et al. demonstrated that approximations of these parameters are feasible by applying live cell microscopy techniques to routine duodenal biopsies, allowing simultaneous assessment of neuronal calcium responses and mitochondrial membrane potential in enteric neurons from Parkinson disease (PD) patients and controls [34].

#### Oxidative damage

Reactive oxygen species (ROS) are defined as unstable, highly reactive molecules containing oxygen with the capacity to cause damage to cellular macromolecules. Under physiological conditions, mitochondria generate ROS during oxidative phosphorylation as byproducts of ATP production, with approximately 1–2% of consumed oxygen being converted into free ROS [35]. While these oxidative species are mostly associated with detrimental effects, they also serve important functions in cell signaling and, specifically in neurons, contribute to neuronal polarity, cytoskeletal modifications, growth cone pathfinding, connectivity, and structural plasticity [36–40]. However, when mitochondria become dysfunctional, ROS levels rise sharply. This creates an imbalance between ROS production and the cell’s antioxidant defenses, a state known as oxidative stress [41]. In general, neurons are particularly susceptible to oxidative stress due to their higher energy demand and O_2_ consumption, abundant mitochondria, relatively poor antioxidant defenses, and limited duplication potential [42]. mtDNA is especially susceptible to oxidative damage because of its close proximity to ROS production sites and the limited repair mechanisms within mitochondria themselves (see below). ROS-induced mtDNA damage impairs mitochondrial function, further increasing ROS production and creating a vicious cycle that likely contributes to neurodegeneration [43]. In the CNS, oxidative stress and mitochondrial dysfunction are strongly associated with neuronal loss in a broad spectrum of neurodegenerative diseases (discussed in more detail later) [44–46].

Similar to the CNS, enteric neurons exhibit high sensitivity to oxidative stress, which has been shown to alter neuronal electrophysiological properties, damage neuronal membranes, and trigger neuronal death [47–49]. Moreover, myenteric neurons, which control gut motility, appear to exhibit a high density of mitochondria, which may explain their susceptibility to increases in ROS production in pathological conditions [50]. Experimental models provide supporting evidence for the role of oxidative stress in ENS dysfunction. In chemically induced colitis, for instance, administration of dinitrobenzene sulfonic acid triggers inflammation in the colon accompanied by increased ROS levels in enteric glia and enteric neuronal loss. Notably, this neuronal damage can be mitigated by the antioxidant N-acetylcysteine, indicating a causal involvement of ROS in enteric neurodegeneration [47]. Moreover, a genetic model of spontaneous chronic colitis demonstrated widespread neuropathy in the ENS, along with elevated mitochondrial superoxide, oxidized DNA adducts, and translocation of high mobility group box 1 (HMGB1) from enteric neurons [51]. These markers collectively indicate that mitochondrial ROS drives neuronal damage in chronic intestinal inflammation [51]. Lastly, McQuade et al. investigated the effect of oxaliplatin, a chemotherapeutic agent known to induce oxidative stress and GI side effects, in Balb/c mice. They found that oxaliplatin treatment resulted in mitochondrial membrane depolarization and significant neuronal loss in both the submucosal and myenteric plexus layers. This study offered direct mechanistic evidence linking mitochondrial dysfunction and ROS overproduction to enteric neurodegeneration and impaired colonic motility [52]. Together, these findings highlight oxidative stress as a central driver of ENS dysfunction.

#### Calcium overload

Calcium is a highly versatile and tightly regulated intracellular messenger that mediates numerous neuronal processes, from excitability and neurotransmitter release to gene transcription and programmed cell death [53]. In neurons, the regulation of intracellular calcium levels relies heavily on mitochondria, which, along with the endoplasmic reticulum (ER), act as major calcium-buffering organelles. Mitochondria can accumulate substantial amounts of calcium, allowing them to respond dynamically to fluctuations in cytosolic calcium [54]. More specifically, under resting conditions, the calcium concentration inside mitochondria is around 100–200 nM, while during stimulation with calcium-increasing agents, mitochondria accumulate 10- to 20-fold more calcium than the cytosolic compartment [55]. Upon neuronal activation, calcium enters the cytosol from the extracellular space through plasma membrane channels, but the consequent increase in free cytosolic calcium is strongly modulated by uptake and release mechanisms of the ER and mitochondria. In particular, mitochondrial calcium uptake occurs via the mitochondrial calcium uniporter (MCU), a calcium-sensitive channel that, when opened by elevated cytosolic calcium, allows calcium to flow into the matrix down the mitochondrion’s steep electrochemical gradient [56, 57].

In neurons, this dynamic calcium buffering system enables mitochondria to regulate a wide array of neuronal functions. Physiologically, intramitochondrial calcium helps match metabolic output to neuronal activity by promoting ATP synthesis. It also plays crucial roles in shaping local and global calcium signals, thereby influencing synaptic transmission, excitability, and neuronal plasticity [58, 59]. Furthermore, mitochondrial calcium participates in retrograde signaling to the nucleus, regulation of organelle trafficking and dynamics, and modulation of ROS production. However, this beneficial role of mitochondrial calcium can shift toward pathological when homeostasis is disrupted. Excessive or sustained mitochondrial calcium uptake, a condition often referred to as mitochondrial calcium overload, can trigger adverse downstream consequences. For example, calcium overload sensitizes mitochondria to permeability transition pore (mPTP) opening, leading to membrane depolarization, release of pro-apoptotic factors, and subsequent cell death [60]. In neurons, where precise calcium handling is vital for maintaining excitatory-inhibitory balance and synaptic integrity, dysregulation of mitochondrial calcium transport is a well-recognized contributor to neurodegenerative processes, such as seen in Alzheimer disease (AD) [61].

In the ENS, emerging evidence points to similar neuronal cell vulnerabilities. A study by Delfino et al. characterized mitochondria-associated ER membranes (MAMs) in the enteric neurons of wild-type C57BL/6J and senescence-accelerated SAMP8 mice [62]. They found widespread MAM structures in enteric neurons and observed disrupted ER-mitochondria interactions in the SAMP8 mice, a model used to recapitulate accelerated aging phenotypes. Functional assays in primary enteric neuron cultures revealed significant alterations in calcium homeostasis in SAMP8 animals, providing the first direct evidence of impaired ER-mitochondria calcium handling in the ENS during neurodegeneration [62]. These findings suggest that, much like in the CNS, mitochondrial calcium dysregulation contributes to enteric neuronal vulnerability with aging and neurodegenerative diseases.

#### Cell death

Several pathological triggers, of which excessive intracellular calcium and oxidative stress are the most prominent, can initiate or contribute to mitochondrial dysfunction. Classically, these stressors are sensed by mitochondria and result in the opening of the mPTP, a non-specific channel in the inner mitochondrial membrane [63–65]. As noted above, opening of the mPTP is primarily induced by elevated mitochondrial matrix calcium levels, in combination with oxidative stress, ADP/ATP imbalance, or phosphate accumulation, all of which are hallmarks of metabolic stress. During metabolic distress, the opening of the mPTP disrupts the proton electrochemical gradient, collapses the mitochondrial membrane potential, and alters the pH gradient, collectively compromising mitochondrial integrity. This increase in permeability not only accelerates ROS accumulation, propagating oxidative damage through lipid peroxidation and membrane destruction, but also leads to leakage of pro-apoptotic factors into the cytosol [66]. One of the key proteins released is cytochrome c, which, under normal conditions, functions as an electron carrier in the mitochondrial respiratory chain. However, upon release, cytochrome c binds to apoptosis-activating factor-1 to form the apoptosome, initiating the intrinsic apoptotic cascade. This cascade activates executioner caspases, leading to the cleavage of cellular components and eventual neuronal apoptosis [67].

Several forms of regulated cell death contribute to neurodegeneration. Apoptosis is the most studied mechanism, providing a controlled pathway of neuronal elimination, but necroptosis and ferroptosis have also emerged as important contributors in various CNS pathologies. Unlike apoptosis, necrosis is a caspase-independent pathway that results in plasma membrane rupture and inflammation, while ferroptosis is an iron-dependent form of cell death characterized by overwhelming lipid peroxidation [68]. In addition, parthanatos represents a distinct form of cell death, initiated by hyperactivation of the DNA strand-break response protein, poly(ADP-ribose) polymerase-1 (PARP1), in reply to excessive genomic stress. This hyperactivation leads to metabolic disturbances, the accumulation of PAR polymers and the release of apoptosis-inducing factors from the mitochondria that translocate to the nucleus and trigger DNA fragmentation [69]. Parthanatos-mediated cell death is activated in certain types of CNS injury and implicated in aggravating neuronal damage, and it can also intersect with other death pathways, such as apoptosis and mitophagy [70]. Neuronal loss underlies a range of physical and cognitive impairments, depending on which neurons are lost and the extent of the damage, thereby driving neurodegenerative diseases [71, 72].

Several studies have provided evidence that enteric neurons, similar to CNS neurons, can undergo apoptosis under pathological conditions. For instance, Bassotti et al. demonstrated that patients with idiopathic slow transit constipation exhibit a significant reduction in enteric neurons, glial cells, and interstitial cells of Cajal, outcomes that correlated with an increased prevalence of apoptotic enteric neurons as assessed by TUNEL staining [73]. Similarly, in irritable bowel syndrome (IBS), apoptosis has been reported in both enteric neurons and glia, with increased neuronal apoptosis in Crohn disease and altered patterns in ulcerative colitis, detected via immunohistochemical assays distinguishing apoptosis from necrosis [74]. Further supporting apoptosis as a mechanism of enteric neurodegeneration, Fan et al. found that sera containing anti-enteric neuronal antibodies from IBS patients induced caspase-3 activation and TUNEL positivity in cultured myenteric neurons, suggesting that antibody-mediated apoptotic pathways contribute to neuronal loss in a subset of IBS patients [75]. Taken together, these findings indicate that apoptosis is a prevalent form of regulated cell death in the ENS across diverse disease contexts.

#### Mitochondrial resilience pathways

**Fig. 2:**
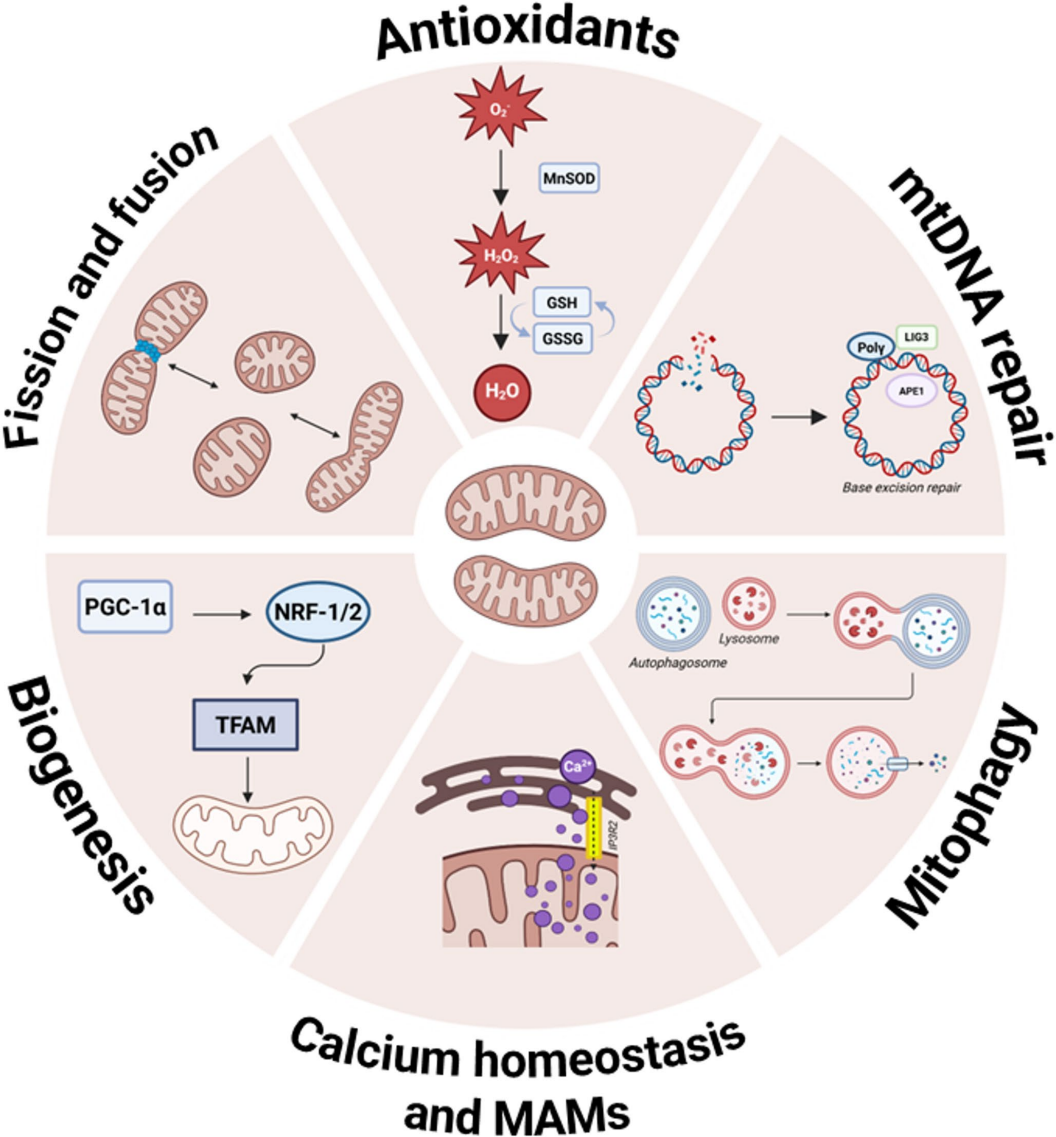
***Overview of mitochondrial resilience pathways.*** APE1, apurinic/apyrimidinic endonuclease 1; GSH, reduced glutathione; GSSG, oxidized glutathione; IP3R2, inositol 1,4,5-trisphosphate receptor type 2; LIG3, DNA ligase III; MAMs, mitochondria-associated membranes; mtDNA, mitochondrial DNA; MnSOD, manganese superoxide dismutase; NRF-1/2, nuclear respiratory factor 1/2; PGC-1α, peroxisome proliferator–activated receptor gamma coactivator 1-alpha; Polγ, DNA polymerase gamma; TFAM, mitochondrial transcription factor A

Fortunately, cells are equipped with protective mechanisms to prevent or counteract unavoidable (macro)molecular damage to mitochondria. These systems include antioxidant defenses and a suite of mitochondrial quality control processes, namely mitochondrial dynamics (fusion and fission), mitophagy, and biogenesis. Together, these processes help maintain mitochondrial integrity, support neuronal survival, and limit dysfunction during disease or stress. Figure 2 summarizes the main resilience pathways that are discussed below. Other adaptive pathways, such as mitohormesis, the mitochondrial unfolded protein response, and the integrated stress response, also contribute to mitochondrial resilience. However, these mechanisms are discussed extensively elsewhere and fall beyond the scope of this review [76].

#### Antioxidants

Neurons are particularly susceptible to oxidative damage due to their relatively weak antioxidant defenses. This vulnerability is exacerbated by their high metabolic activity and reliance on mitochondrial respiration, which inevitably produces ROS as byproducts [17]. Antioxidants play a critical role in mitigating oxidative stress by scavenging free radicals that initiate macromolecular damage, particularly lipid peroxidation, and preventing the propagation of the radical chain, in which lipid radicals continuously generate new radicals in adjacent membrane lipids, exacerbating cellular injury [77]. Among the antioxidant defenses, glutathione (GSH) plays a central role as the primary redox buffer, detoxifying hydrogen peroxide via glutathione peroxidase and maintaining the reduced state of proteins critical for neuronal survival. A reduction in intracellular GSH leads to an increase in mitochondrial ROS levels and depolarization of the mitochondrial membrane, a feature of mitochondrial dysfunction [78]. Although at low levels, neurons do express enzymatic antioxidants as well, such as superoxide dismutases (SODs), catalase, and peroxiredoxins. SOD1 and SOD2, which convert superoxide radicals into hydrogen peroxide, are especially important, with the former residing within both the cytosol and the mitochondrial intermembrane space, and the latter located exclusively within the mitochondrial matrix [79]. It has been shown that pathogenetic variants in *SOD1* are directly linked to amyotrophic lateral sclerosis (ALS), a progressive, fatal neurodegenerative disorder (see later), underscoring the importance of superoxide detoxification in neuronal health [80]. Catalase and glutathione peroxidase further detoxify hydrogen peroxide into water, while peroxiredoxins provide additional layers of ROS regulation [79].

Redox regulation is central to ROS-mediated signaling and mitochondrial function. Given the significance of proper redox homeostasis, mitochondria-targeted antioxidants have been explored as therapeutic strategies in neurodegenerative disease. MitoQ, a ubiquinone derivative conjugated to a lipophilic triphenylphosphonium cation that drives mitochondrial accumulation, has been extensively studied. In a mouse model of ALS, MitoQ slowed mitochondrial functional decline in the spinal cord and muscle, decreased nitroxidative damage in the nervous system, delayed symptom progression, and extended survival [81]. In the Huntington disease (HD) R6/2 mouse model, MitoQ ameliorated fine motor impairments by reducing oxidative damage in muscle tissue; its bioavailability in the brain was limited, highlighting tissue-specific effects [82]. Another promising approach is XJB-5–131, a bifunctional compound combining a nitroxide radical scavenger with a mitochondrial-targeting moiety. In HD models, XJB-5–131 improved mitochondrial function, suppressed disease phenotypes, and enhanced neuronal survival, demonstrating the therapeutic potential of mitochondria-targeted radical scavengers. Quercetin, while not mitochondria-specific, is a potent antioxidant with reported benefits in conditions of enteric oxidative stress [83]. A study by Sehaber-Sierakowski et al. demonstrated that administration of quercetin-loaded microcapsules, which are antioxidants designed for controlled release in the gut, reduced oxidative stress in the ileum of adult diabetic Wistar rats. However, this treatment did not prevent overall neuronal loss in the ENS [84]. Nonetheless, these early findings suggest that antioxidant-based strategies may hold therapeutic potential, particularly for IBS where excessive ROS production and oxidative stress are central to disease pathology.

#### DNA repair

Mitochondrial DNA (mtDNA) is particularly vulnerable to damage due to its proximity to the electron transport chain, where ROS are continuously generated. To counteract such damage, mitochondria have several DNA repair pathways, including versions of direct reversal, mismatch repair, and double-strand break repair [85]. However, the most prominent and well-characterized pathway in mitochondria is base excision repair (BER). The primary function of BER is to remove oxidized, alkylated, or deaminated bases, and select mitochondria-targeted isoforms (via alternative splicing or translation) often exist for many of the classic BER proteins [86]. Similar to nuclear BER, in mitochondrial BER, upon damage recognition, base excision occurs via a DNA glycosylase, such as uracil-DNA glycosylase (UDG), 8-oxoguanine glycosylase (OGG1), or endonuclease III-like protein 1 (NTH1), resulting in apurinic/apyrimidinic (AP) site products that are processed mainly by AP endonuclease 1 (APE1). The resulting 1-nt gap is then filled by the major mitochondrial DNA polymerase (Polγ), although evidence supports contributions by Polβ as well. Final nick sealing is mediated by a mitochondrial version of DNA ligase III (LIG3), which is essential in mitochondria but compensated for in the nucleus by LIG1 and LIG4. Together, these steps of BER maintain mtDNA integrity and preserve mitochondrial function [87, 88].

Notably, several independent studies have reported that impaired BER contributes to neuronal vulnerability in both CNS and ENS disorders [33, 89]. For example, Imam et al. showed that incision activities of multiple mitochondrial glycosylases (OGG1, UDG, and NTH1) decline across several brain regions in aging mice, whereas nuclear repair exhibited more variable changes. This suggests that reduced mitochondrial BER efficiency contributes to the accumulation of oxidative mtDNA damage during aging, thereby increasing mtDNA mutation burden and possibly promoting neuronal loss and age-related neurodegeneration [90]. Moreover, an imbalance in BER components makes neurons more vulnerable to oxidative stress as shown by Harrison et al. They reported that primary cerebellar granule cells, compared with astrocytes, exhibit less efficient repair of mtDNA lesions and increased vulnerability to oxidative stress–induced apoptosis. Interestingly, these neurons displayed elevated APE1 activity but reduced Polγ activity, pointing to a mismatch between lesion recognition/incision and gap-filling mechanisms. This imbalance increases the persistence of mtDNA damage, linking BER dysfunction directly to mitochondrial-driven apoptosis in neurons [91]. In the context of the ENS, as expanded upon later, Bonora et al. found that pathogenetic variants in *LIG3* cause mitochondrial neurogastrointestinal encephalomyopathy (MNGIE)-like syndromes with CIPO [33]. Lastly, mtDNA genome maintenance is intertwined with mitochondrial dynamics, as fusion events allow for functional complementation between mitochondria by mixing mtDNA and proteins, thereby buffering against high levels of mtDNA damage [92].

#### Fission and fusion

Mitochondria are highly dynamic organelles that continuously undergo fission and fusion to maintain their morphology, distribution throughout the cell, and function. These structural adaptations are crucial for key cellular processes, including cell cycle progression, immune responses, apoptosis, and mitochondrial quality control. The balance between fission and fusion is tightly regulated by GTPases and is essential for maintaining cellular homeostasis, especially in post-mitotic cells, such as neurons [93]. Mitochondrial fusion consists of the joining of two mitochondria, enabling the mixing of contents to mitigate existing damage in some cases. This process is mediated by mitofusins 1 and 2 (MFN1 and MFN2) on the outer mitochondrial membrane and by optic atrophy 1 (OPA1) on the inner membrane [94]. Through fusion, essential functional and structural components, such as proteins, lipids and mtDNA, in two mitochondria can be mixed and diffused, thereby counteracting the effects of stress and rescuing the dysfunctional mitochondria [95–98]. In contrast, mitochondrial fission, primarily driven by dynamin-related protein 1 (DRP1) and its adaptor, fission 1 homologue (FIS1), results in the segregation of damaged mitochondrial components [99]. The separated, damaged fragments can then be selectively targeted for degradation via mitophagy, thus preserving mitochondrial quality. Fission also has a role in mitochondria proliferation during cell division, helping to retain a healthier mitochondrial population [100].

In response to oxidative stress, fusion and fission dynamics are altered to preserve cellular energy demands and limit damage. Mitochondrial damage primarily results in ATP depletion, lowering the ATP/AMP ratio. This decreased ratio activates AMP-activated protein kinase (AMPK), an essential energy sensor, which in turn inhibits the mammalian target of rapamycin complex 1 (mTORC1), a crucial positive regulator of mitochondrial metabolism and biogenesis [101]. However, when mitochondrial damage exceeds resilience capacity, mitochondria may rupture and release apoptotic factors that ultimately cause cell death. This is particularly detrimental for postmitotic neurons, including those of the CNS and ENS.

In the CNS, fusion and fission dynamics are not only stress responses but also essential regulators of neuronal physiology. Pathogenetic variants in core regulators such as *OPA1* and *MFN2* cause Charcot-Marie-Tooth neuropathy, a group of inherited, progressive genetic disorders that involve damage to the peripheral nerves, highlighting the importance of mitochondrial dynamics for neuronal integrity [102, 103]. Furthermore, Cagalinec et al. demonstrated that fusion–fission cycles tightly regulate mitochondrial morphology and motility in neurons [104]. They also reported that pathological conditions, such as overexpression of mutant Huntingtin or Tau protein, impair mitochondrial motility, suppress fusion, and lead to mitochondrial fragmentation. Remarkably, restoring motility via Miro-1 overexpression in the aforementioned pathological cell models rescued fusion and normalized mitochondrial morphology [104]. Altogether, the findings underscore how dysregulated mitochondrial dynamics contribute to neurodegenerative processes in the CNS. In the ENS, direct evidence is more limited, but recent studies point toward similar vulnerabilities. Patient-derived fibroblasts from individuals with CIPO have shown altered expression of fission and fusion markers, suggesting that disrupted mitochondrial dynamics may underlie enteric neuronal dysfunction [105].

#### Mitophagy

Mitophagy is a specialized form of autophagy that targets dysfunctional mitochondria for degradation in lysosomes, playing a critical role in mitochondrial quality control and cellular homeostasis [106]. This process ensures the preservation of mitochondrial function and integrity. Irreparably damaged mitochondria, often characterized by depolarized membranes or heavily mutated mtDNA, are recognized and targeted for lysosomal degradation. During the process, the dysfunctional mitochondria are sequestered into double-membrane autophagosomes, which subsequently fuse with lysosomes for degradation and recycling of their content [107]. In neurons, two primary mitophagy pathways have been characterized: the ubiquitin-mediated and receptor-mediated pathways. The ubiquitin-mediated pathway is primarily regulated by PTEN-induced putative kinase 1 (PINK1) and the E3 ubiquitin ligase Parkin. Upon mitochondrial depolarization, PINK1 accumulates on the outer mitochondrial membrane and recruits cytosolic Parkin, two proteins that are defective in familial forms of PD (see later). Parkin ubiquitinates various outer membrane proteins, marking the damaged mitochondrion for autophagic clearance [108–110]. The receptor-mediated pathway involves mitophagy receptors located on the mitochondrial membrane that contain LC3-interacting regions (LIRs). These receptors, such as BCL2 interacting protein 3 (BNIP3), FUN14 domain containing 1 (FUNDC1), and prohibitin 2 (PHB2), directly interact with LC3, resulting in autophagosome recruitment and mitochondrial engulfment [111–113].

Multiple reports suggests a close interplay between mitophagy and mitochondrial dynamics. For example, mitochondrial fission appears to occur before mitophagy, as it allows the segregation of damaged mitochondrial regions prior to their degradation. Moreover, studies by Twig and Yu et al. show that overexpression of OPA1 or inhibition of DRP1-mediated fission can suppress mitophagy, and components of the PINK1/Parkin pathway have been shown to interact with the fission/fusion machinery [114, 115]. Mitophagy is particularly crucial in neurons, where it maintains cellular health by removing dysfunctional mitochondria that would otherwise generate excessive ROS and impair neuronal viability. Importantly, impaired mitophagy has been implicated in both CNS and ENS disorders, including PD, AD and CIPO, underscoring its essential role in neuronal survival [105, 116].

#### Biogenesis

Mitochondrial biogenesis is a tightly regulated cellular process essential for maintaining mitochondrial function and adapting to metabolic demands or stress. It involves the generation of new mitochondria to replace damaged ones, expansion of the mitochondrial network, and an increase in energy production capacity. This process is particularly important in cells with high energy requirements, such as neurons [117]. Mitochondrial damage triggers signaling cascades that activate redox-sensitive transcriptional programs promoting mitochondrial biogenesis. Central to this response is peroxisome proliferator-activated receptor gamma coactivator 1-alpha (PGC-1α), which regulates transcriptionally the expression of nuclear-encoded mitochondrial genes, including those involved in oxidative phosphorylation and antioxidant defense. PGC-1α coactivates nuclear respiratory factors, namely nuclear respiratory factor 1 and 2 (NRF-1 and NRF-2), which in turn upregulate mitochondrial TFAM, a key protein responsible for mtDNA replication and transcription. During biogenesis, new mitochondrial proteins are synthesized in the cytosol, while lipids are recruited from cellular pools, and together the two elements are assembled into functional complexes. Simultaneously, mtDNA is replicated to ensure sufficient genetic material. Mitochondrial fission separates the newly formed mitochondria from the existing network. These new mitochondria are then functionally integrated into the expanding mitochondrial network, thereby restoring or enhancing the cell’s bioenergetic capacity [118].

In neurons, this process is tightly linked to synaptic activity, as activity-dependent calcium signaling can stimulate PGC-1α pathways to boost mitochondrial content in dendrites and axons, thereby sustaining synaptic transmission and plasticity [119]. Knockdown of PGC-1α results in a marked decrease of dendritic mitochondria and a reduction in the number of synapses, an effect observed in both embryonic neurons and adult brains. This finding underscores the critical role of PGC-1α not only in synapse formation but also in long-term maintenance, consistent with the broader requirement of mitochondria for filopodia formation and axonal branch maturation [119, 120]. Conversely, impaired biogenesis, as reported in neurodegenerative diseases such as AD and PD, contributes to reduced mitochondrial mass and function, exacerbating neuronal vulnerability [121, 122]. Thus, mitochondrial biogenesis acts not only as a general quality control mechanism but also as a neuron-specific adaptive response to maintain synaptic health and resilience under stress.

### Mitochondria-associated membranes and calcium homeostasis

As mentioned earlier, mitochondria play a central role in calcium buffering, a process that is closely coordinated at MAMs, which are specialized contact sites where the ER and mitochondria are in close proximity [123]. MAMs are composed of key structural and regulatory proteins, such as MFN2, inositol 1,4,5-trisphosphate receptor (IP3R), voltage-dependent anion channel 1 (VDAC1), glucose-regulated protein 75 (GRP75), and phosphofurin acidic cluster sorting protein 2 (PACS-2). These proteins form a macromolecular complex, IP3R-GRP75-VDAC1, that serves as a conduit for calcium transfer from the ER lumen to the outer mitochondrial membrane. From there, calcium is transported into the mitochondrial matrix via the MCU on the inner mitochondrial membrane, driving ATP synthesis. Beyond their metabolic and signaling roles, MAMs have gained increasing attention for their contribution to neuronal health and disease [124]. These contact sites regulate autophagy, phospholipid exchange, and stress responses, and their dysregulation has been linked to age-related neurodegeneration in the CNS, including in AD and PD [125–127]. Recently, the relevance of MAMs has also been extended to the ENS. The previously mentioned study by Delfino et al. provided the first ultrastructural and functional characterization of MAMs in enteric neurons, showing that while MAMs are abundant in the ENS, their integrity is compromised in senescence-accelerated mouse models, such as the SAMP8 mice. These neurons displayed altered calcium handling, as observed with mitochondrial calcium imaging, and impaired mitochondrial-ER communication, mirroring defects observed in CNS models of neurodegeneration [62]. This suggests that MAM disruption may similarly impair enteric neuronal function, potentially contributing to the GI symptoms often observed in neurodegenerative diseases.

#### Mitochondrial dysfunction in neurodegenerative disorders

The selective and progressive loss of neuronal cells is a hallmark of neurodegenerative disorders, leading to the gradual deterioration in CNS and/or ENS function. Despite involving distinct neuronal populations, neurodegenerative diseases share common traits, e.g., severe neuronal cell loss, impaired axonal transport, and the accumulation of dysfunctional mitochondria. Moreover, failure of mitochondrial quality control is expected to exacerbate or even initiate several CNS disorders, including AD and PD. Similarly, in the ENS, mitochondrial dysfunction has been linked to disorders such as MNGIE. Table 1 summarizes the multifaceted systemic consequences of mitochondrial dysfunction in degenerative diseases, which we now cover as primary or secondary disorders based on the type and extent of mitochondrial defects. As classically defined, we consider primary mitochondrial disorders as diseases that stem from loss of or pathogenic variants in mitochondrial or nuclear genes that encode proteins essential for oxidative phosphorylation and energy production [i.e., electron transport chain (ETC) genes or gene products that are responsible for ETC complex formation]. Such disorders predominantly affect high-energy organs, e.g., brain, muscle and heart. Secondary mitochondrial disorders are considered those that involve mitochondrial dysfunction that arises from factors besides directly impaired oxidative phosphorylation, such as pathogenic variants in mitochondrial-related resilience mechanisms, disease-specific pathological events (e.g., protein aggregation), oxidative stress, or exposure to harmful external agents, often manifesting in broader systemic clinical phenotypes [128].

**Table 1:** Neurogenerative disorders exhibiting mitochondrial dysfunction and associated brain, motor or gastrointestinal (GI) symptoms

	Brain Symptoms	Motor Symptoms	GI Symptoms	Ref
Primary mitochondrial diseases				
Mitochondrial neurogastrointestinal encephalomyopathy	Leukoencephalopathy, peripheral neuropathy	Myopathy, ptosis	Severe GI dysmotility, chronic intestinal pseudo-obstruction	[215]
Secondary mitochondrial diseases				
Parkinson Disease	Cognitive decline (late), depression, sleep disturbances, apathy, anhedonia	Tremor, muscle rigidity, bradykinesia	Constipation, postprandial fullness, and gastric retention	[216]
Alzheimer Disease	Memory loss, confusion, disorientation	Bradykinesia, rigidity, tremor	Constipation, gut-brain axis changes	[217, 218]
Huntington Disease	Cognitive decline, psychiatric symptoms	Chorea, dystonia, motor incoordination	Weight loss, dysphagia, altered GI motility	[219, 220]
Amyotrophic Lateral Sclerosis	Cognitive and behavioral changes in some cases	Progressive weakness, spasticity, muscle atrophy, paralysis	Early intestinal dysmotility, constipation, impaired ENS function, microbiome alterations	[221–223]

#### Primary mitochondrial disorders

##### Mitochondrial neurogastrointestinal encephalomyopathy

MNGIE is a rare genetic primary mitochondrial disorder that involves progressive GI failure, nerve/muscle wasting, and premature death. Classic MNGIE stems from pathogenetic variants in the *TYMP* gene, which encodes thymidine phosphorylase, an enzyme crucial to the nucleotide salvage pathway that generates nucleotides for reuse in DNA synthesis. Loss of thymidine phosphorylase activity causes accumulation of thymidine and deoxyuridine, leading to an imbalance in mitochondrial nucleotide pools. This nucleotide imbalance disrupts replication and repair of mtDNA, causing deletions and genome depletion that results in impaired oxidative phosphorylation and consequent energy failure [129]. Recently, Bonora et al. identified a novel form of MNGIE associated with biallelic pathogenic variants in *LIG3* across three unrelated families that also presented with CIPO as a central clinical feature [33]. Other well-known primary mitochondrial disorders, which will not be covered further herein, include: mitochondrial encephalomyopathy, lactic acidosis, and stroke-like episodes (MELAS); myoclonic epilepsy with ragged-red fibers (MERRF); Leber hereditary optic neuropathy (LHON); and Kearns-Sayre (eye muscle weakness, heart issues) and Leigh (severe neurological decline) syndromes [130].

As alluded to earlier, the human genome encodes three separate DNA ligases (I, III, and IV), all of which are present within the nucleus. In the nucleus, LIG3 functions in the BER pathway, typically in complex with the single-strand break repair protein, X-ray repair cross-complementing protein 1 (XRCC1). As alluded to earlier, LIG3 is uniquely located in mitochondria, transported there as a mitochondrial-targeted isoform, i.e., LIG3α, which is produced via alternative splicing and contains an N-terminal mitochondrial targeting sequence [131]. While the loss of LIG3 in the nucleus can be generally compensated by the other DNA ligases, mitochondrial LIG3 is indispensable for mtDNA replication and repair [132, 133]. Thus, reduced LIG3 activity is expected to preferentially compromise mtDNA status and mitochondrial health, leading to conditions linked to oxidative phosphorylation defects and mitochondrial dysfunction. Interestingly, enteric neurodegeneration has been reported in a MNGIE-CIPO patient harboring *LIG3* pathogenetic variants [134]. Fibroblasts derived from these CIPO-LIG3 patients exhibit multiple hallmarks of mitochondrial impairment: abnormal cristae and swollen mitochondria with increased average area, as observed with transmission electron microscopy, as well as reduced uncoupled oxygen consumption and decreased ATP levels. These cells also showed an elevated production of mitochondrial ROS relative to controls, supporting the existence of mitochondrial dysfunction [33]. Morphological analysis revealed an altered mitochondrial network as well. This was accompanied by a reduction in OPA1 and MFN2 protein levels, indicating disrupted mitochondrial dynamics, particularly fission/fusion and mitophagy. In addition to the structural and metabolic deficits, LIG3-mutant fibroblasts displayed dysregulated mitochondrial calcium homeostasis. More specifically, calcium imaging studies of the fibroblasts revealed reduced mitochondrial calcium levels, and investigation of the MAM tethering complex uncovered a significant reduction in IP3R expression, while levels of GRP75, VDAC1, and MCU remained unchanged [105]. This suggests that impaired ER-mitochondrial calcium signaling may contribute to the disease phenotype, namely the gut fibrosis observed in LIG3-related CIPO [105].

#### Secondary mitochondrial disorders

While there have been arguments made that the following neurodegenerative disorders are driven by mitochondrial dysfunction, the mitochondrial and energy deficits observed in these so-called secondary diseases primarily arise downstream of indirect pathogenic mechanisms, such as disruption of relevant quality control pathways, oxidative stress, or other means.

##### Parkinson disease

PD is characterized by the progressive degeneration of dopaminergic neurons in the substantia nigra pars compacta and the pathological accumulation of α-synuclein in Lewy bodies. Given the extreme reliance of this specialized type of neuron on mitochondrial oxidative phosphorylation for ATP production, it is not surprising that secondary mitochondrial dysfunction is a central and consistent hallmark of both idiopathic and familial forms of PD [135]. Notably, deficits in complex I of the electron transport chain, elevated oxidative stress, and impaired mitochondrial quality control mechanisms, such as mitophagy, fission and fusion, are commonly observed in PD brains [136–139].

In genetic forms of PD, several familial PD genes encode proteins that play a role in mitochondrial pathways: DJ-1, a mitochondrial kinase, is a stress-response molecular chaperone that supports complex I, and Parkin works with PINK1 to regulate mitochondrial morphology, dynamics, and turnover. Pathogenetic variants in these genes disrupt mitochondrial function, leading to reduced ATP production, increased ROS levels, and ultimately neuronal loss [140–142]. Additionally, α-synuclein itself contributes to mitochondrial damage by interfering with complex I activity, disrupting mitochondrial membrane potential, and promoting ROS production. Many of the PD associated proteins have also been implicated in the regulation of mitochondrial stress responses and the maintenance of calcium and ROS homeostasis [45, 143–147]. A study in *Drosophila* has shown that loss of Pink1 or Parkin results in enlarged mitochondria, suggesting impaired fission, while overexpression of Parkin can rescue the mitochondrial defects caused by Pink1 loss [148]. In mammalian models, loss of PINK1 kinase function leads to fragmented and truncated mitochondria, a phenotype that can be rescued by engineered Parkin expression. This shows that the PINK1–Parkin pathway is an evolutionarily conserved mechanism [149, 150].

Beyond the classic motor symptoms, PD is also associated with GI issues, including constipation, which has led to the hypothesis that the ENS may be an early site of PD pathology. While the presence of aggregated α-synuclein in the CNS has been well established, it is unclear whether this harmful event also occurs in the ENS. However, a study by Corbillé et al. showed that α-synuclein exists primarily as a monomer in primary cultures of rat enteric neurons, suggesting that the state of α-synuclein varies between the ENS and CNS [151]. In human-based studies, evidence has been mixed. One study using live calcium and mitochondrial imaging in submucosal enteric neurons from duodenal biopsies found no significant differences between PD patients and controls in terms of calcium signaling, mitochondrial membrane potential, mitochondrial volume, or neuronal density [34]. Furthermore, α-synuclein staining was comparable between PD and control groups, suggesting that submucosal neurons may remain functionally intact even in PD patients experiencing GI symptoms. This challenges the idea that submucosal dysfunction initiates PD and instead implicates other regions, such as the myenteric plexus or brainstem nuclei as more critical to early disease progression. Conversely, another study using autopsy material did offer evidence for mitochondrial involvement at the enteric level [152]. By analyzing ganglia from PD patients without GI symptoms and age-matched controls, Baumuratov et al. found that submucosal colon ganglia from PD patients exhibited smaller volume, a significantly higher number of mitochondria per ganglion volume, and an overall increase in mitochondrial mass compared to controls. These findings suggest that mitochondrial accumulation could reflect a compensatory response aimed at maintaining neuronal function or simply failed removal [152].

##### Alzheimer disease

AD is a progressive neurodegenerative disorder marked by memory loss, cognitive decline, and behavioral changes. Its pathology has been described along the ATN framework, in which amyloid-β (Aβ) deposition (A), Tau aggregation (T), and neurodegeneration (N) together define disease progression. According to the long-standing Amyloid Cascade Hypothesis, the early buildup of Aβ, the primary component of amyloid plaques, initiates a cascade of pathological events that ultimately lead to neuronal damage [153]. However, growing evidence challenges this model, as Aβ burden does not consistently correlate with cognitive decline, particularly in the patients older than 85, where post-mortem studies often show weak associations between plaque load and clinical symptoms [154, 155]. Contrary to the Amyloid Cascade Hypothesis, the Mitochondrial Cascade Hypothesis (MCH) has been proposed, arguing that mitochondrial dysfunction is not just a downstream effect but may be a central driver of AD pathogenesis. According to this model, inherited or age-related mitochondrial deficits lead to bioenergetic decline, increased ROS production, and impaired mitochondrial quality control. These mitochondrial alterations then accelerate Aβ and Tau pathology, ultimately driving neurodegeneration [156]. While still controversial and less widely accepted than the ATN model, the MCH underscores the possibility that mitochondria play a more central role in AD than traditionally proposed.

Regardless of whether mitochondrial changes are a cause or a consequence of disease manifestation, mitochondrial impairment is a consistent hallmark of AD. This includes the observations of decreased activity of Krebs cycle enzymes, reduced ATP production, and increased oxidative stress in AD biospecimens relative to controls [157–161]. Moreover, cytochrome c oxidase activity is diminished across several cortical regions in post-mortem AD brains [162–164]. Furthermore, PET imaging in early AD stages reveals brain hypometabolism, indicating impaired mitochondrial function and glucose metabolism [165, 166]. Mitochondria are also implicated as intracellular sites of Aβ accumulation; and Aβ, amyloid precursor protein (APP), and APP β-C-terminal fragments have been found in mitochondria-enriched brain fractions of transgenic APP mice [167, 168]. Interestingly, Tasnady et al. demonstrated that Aβ also accumulates in enteric neurons of AD mice, although a direct correlation with mitochondrial dysfunction in the ENS has not been established [169]. Similarly, a study by Lui et al. showed that Aβ mediates intestinal dysfunction and enteric neuron loss in an APP/PS1 AD mouse model, suggesting that the ENS mirrors the neuropathology observed in AD brains [170]. These findings imply that intestinal pathology may represent an early, prodromal event in AD, potentially contributing to the development of CNS symptoms. Notably, neither of the two aforementioned studies investigated whether mitochondrial dysfunction underlies the observed ENS pathology, highlighting an interesting avenue for future research.

In addition, studies show that ER-mitochondrial communication via MAMs is upregulated in AD cell models and in cells from AD patients. Moreover, C99 (the direct precursor of Aβ) was present in MAMs, and the accumulation of C99 may mediate the increased MAM activity and downstream mitochondrial dysfunction. Increases in C99 also induce increased production of ceramide, an inhibitor of mitochondrial respiration and a pro-apoptotic molecule [171].

Tau pathology, the other hallmark of AD, is likewise associated with mitochondrial dysfunction, although the mechanism appears to unfold in a unique manner. In particular, the overexpression of Tau induces perinuclear mitochondrial clustering, altered morphology, increased retrograde trafficking, mitophagy deficits, and reduced Complex I and ATP activity [172–174]. Tau has been specifically localized to the outer and inner mitochondrial membranes and may disrupt ER-mitochondria contact sites [175]. Moreover, Tau-induced stabilization of actin filaments hinders DRP1 translocation to mitochondria, thereby impairing fission and promoting mitochondrial elongation as well as impaired mitophagy [176]. In addition to DRP1, PINK1, a key regulator of mitophagy, is downregulated in AD brains [177]. Consistently, Fang et al. showed reduced mitophagy in multiple AD models, including post-mortem hippocampal tissue, where co-localization of TOMM20 and LAMP2 was decreased, alongside reduced levels of PINK1 and Parkin, broadly supporting impaired mitophagy in AD [116].

Lastly, disrupted calcium homeostasis is observed in AD. Calvo-Rodriguez et al. found increased mitochondrial calcium accumulation in a transgenic mouse model of cerebral β-amyloidosis, a phenotype that was associated with plaque deposition and neuronal loss. Complementary RNA-sequencing data from post-mortem AD brains revealed downregulation of genes encoding mitochondrial calcium influx components, alongside upregulation of efflux pathways, suggesting a compensatory response to chronic mitochondrial calcium overload [61].

##### Huntington disease

Mitochondrial dysfunction is an early and pivotal contributor to the pathogenesis of HD, a neurodegenerative disorder caused by an expanded CAG repeat in the Huntingtin (*HTT*) gene. Characterized by the selective loss of neurons in the striatum and cortex, HD has long been associated with mitochondrial abnormalities. Initial evidence came from ultrastructural changes observed in mitochondria from postmortem HD cortical tissue. Mutant huntingtin (mHtt) protein directly interacts with mitochondrial membranes, disrupting calcium homeostasis, altering morphology, and impairing mitochondrial trafficking. Moreover, it binds to the translocase of the inner mitochondrial membrane, interfering with protein import and bioenergetics, an effect observed even in presymptomatic HD mice [178, 179]. Additionally, mHtt reduces mitochondrial membrane potential by impairing complexes II and III of the respiratory chain, contributing to a progressive energy deficit [180, 181]. Early PET studies showed decreased glucose uptake in the basal ganglia of presymptomatic HD carriers, while elevated lactate levels in HD-affected individuals further underscored mitochondrial metabolic dysfunction [182–185]. Mitochondrial dysfunction in HD also promotes the accumulation of ROS, overwhelming cellular antioxidant defenses and damaging proteins, lipids, and DNA, and mHtt disrupts mitochondrial quality control [186–188]. Moreover, in HD models, PGC-1α levels in medium spiny neurons are drastically reduced, compromising the expression of genes essential for respiration and ROS detoxification [189]. Mitochondrial dynamics are also altered. For example, fusion proteins such as Mfn1, Mfn2, and OPA1 are downregulated, while fission protein Drp1 is excessively recruited to mitochondria [190]. Additionally, calcium handling by mitochondria is severely compromised in HD. More specifically, mHtt enhances ER calcium release via sensitization of IP3 and RyR receptors, while simultaneously reducing the expression of Sig-1R, a chaperone that stabilizes IP3Rs during stress. These alterations reduce calcium buffering capacity and promote early opening of the MPTP, triggering cell death [191–194].

Beyond the effects present in the cortex and striatum, a study by Sciacca et al. found early enteric neuronal dysfunction in mouse and human HD samples. A 56-year-old woman with mild HD developed progressive constipation from age 47, requiring colon resection at 53, just before motor symptoms began. Colon tissue showed accumulation of mHtt and reduced levels of enteric neuropeptides. Similar findings were observed in R6/2 HD mice, where reduction in the same neuropeptides appeared in premanifest stages, and other neuropeptides declined from early symptomatic stages [195]. While these findings highlight that GI dysfunction in HD can precede motor symptoms and may contribute to disease progression, a direct link between enteric defects and mitochondrial dysfunction has yet to be firmly established.

##### Amyotrophic lateral sclerosis

Mitochondrial dysfunction is a central and well-documented feature of ALS, a fatal neurodegenerative disorder characterized by progressive loss of upper and lower motor neurons. Multiple pathogenic mechanisms tie back to mitochondria, including impaired bioenergetics, oxidative stress, defective calcium handling, and disrupted quality control mechanisms [196]. Post-mortem studies and patient-derived models consistently show features of mitochondrial dysfunction, such as swelling, fragmentation, and vacuolization, particularly in motor neurons [197–200]. At the functional level, ALS mitochondria exhibit reduced activity of respiratory chain complexes, diminished ATP production, and increased ROS generation, which together compromise neuronal survival [201–204]. Moreover, genetic factors strongly implicate mitochondria in ALS. For example, pathogenetic variants in *SOD1*, one of the first discovered ALS genes, lead to toxic protein aggregation in the mitochondrial intermembrane space, causing impaired respiration, oxidative damage, and reduced antioxidant defenses [205]. Similarly, pathogenetic variants in ALS-related TAR DNA-binding protein (*TDP-43*) and FUS RNA binding protein (*FUS*) alter mitochondrial dynamics and RNA processing, while *C9ORF72* repeat expansions, another genetic cause of ALS, disturb ER–mitochondria contact sites and calcium transfer [206, 207]. Overall, these genetic defects result in disrupted energy metabolism and defective mitochondrial signaling pathways.

Mitochondrial quality control is also affected in ALS. For instance, proteins regulating fusion and fission, such as OPA1 and DRP1, are dysregulated, leading to imbalanced mitochondrial dynamics [208, 209]. In addition, ALS mitochondria exhibit reduced calcium buffering capacity and increased susceptibility to mPTP opening, ultimately triggering apoptotic and necroptotic pathways [210, 211]. As mentioned earlier, mitochondria-targeted antioxidants have shown promise in experimental ALS models. For example, MitoQ reduced nitroxidative stress, preserved mitochondrial function, and extended survival in ALS mice, while other small molecules aimed at stabilizing calcium homeostasis or promoting mitophagy may offer therapeutic potential as well [81, 212, 213].

Interestingly, findings by Zhang et al. extend mitochondrial involvement in ALS beyond the CNS to the GI tract and ENS. The research team performed longitudinal studies in *SOD1*^*G93A*^ mice and uncovered that intestinal dysmotility, gliosis, and smooth muscle alterations occur before the onset of motor symptoms, revealing a correlation between early mitochondrial dysfunction and aggregation of mutant SOD1 in both the ENS and subsequently the spinal cord. Importantly, manipulation of the gut microbiome using butyrate or antibiotics reduced mutant SOD1 aggregation, improved enteric neuromuscular function, and delayed motor decline. Moreover, transplantation experiments showed that feces from presymptomatic ALS mice accelerated SOD1 aggregation in human colonoids, directly linking microbial dysbiosis to disease pathology [214]. Taken together, ALS shows how mitochondrial dysfunction is involved in oxidative stress, calcium imbalance, and impaired organelle quality control. This mitochondrial pathology not only drives motor neuron degeneration but also highlights mitochondria as an attractive therapeutic target in ALS as well as the other neurodegenerative disorders described above.

## Conclusion

Mitochondria play a fundamental role in neuronal health, influencing bioenergetics, redox balance, calcium homeostasis, and regulated cell death pathways. Their contribution to the onset and progression of neurodegenerative disorders is well established in the CNS, where extensive evidence demonstrates that mitochondrial dysfunction is a major driver of neuronal vulnerability and disease pathology. In contrast, the role of mitochondria in the ENS remains understudied. Nevertheless, given the shared metabolic demands, structural parallels, and functional similarities between CNS and ENS neurons, it is likely that mitochondrial defects contribute to neurodegeneration in the gut as well. Continued investigation into ENS-specific mitochondrial biology will be essential for uncovering disease mechanisms and may ultimately reveal new therapeutic opportunities that span both nervous systems.

## Data Availability

Not applicable.
